# Immunotherapy and Radiation Therapy Combinatorial Approaches in Hepatocellular Carcinoma

**DOI:** 10.3390/cancers16051058

**Published:** 2024-03-05

**Authors:** Alireza Tojjari, James Yu, Anwaar Saeed

**Affiliations:** 1 Division of Hematology & Oncology, Department of Medicine, University of Pittsburgh Medical Center (UPMC), Pittsburgh, PA 15232, USA; alirezatojjari@gmail.com; 2 Division of Hematology and Medical Oncology, H. Lee Moffitt Cancer Center and Research Institute, Tampa, FL 33612, USA; james.yu@moffitt.org

**Keywords:** hepatocellular carcinoma, immune checkpoint inhibitors, immunotherapy, radiation therapy, combination cancer therapies

## Abstract

**Simple Summary:**

In the fight against hepatocellular carcinoma (HCC), the most common form of liver cancer, researchers are exploring the combination of immunotherapy and radiation therapy. This article reviews the latest studies on this approach, focusing on how these two treatments can work together to enhance their effectiveness against HCC. The findings from this research could significantly impact the treatment of liver cancer, potentially leading to more effective strategies and better outcomes for patients.

**Abstract:**

Hepatocellular carcinoma (HCC), a prevalent and often fatal liver cancer, presents significant treatment challenges, especially in its advanced stages. This article delves into the promising approach of combining immunotherapy, particularly immune checkpoint inhibitors, with radiation therapy, a cornerstone of HCC management. Our review synthesizes current preclinical and clinical research, highlighting the potential synergistic effects of this combinational treatment. Emerging evidence suggests that this synergy enhances tumor control and improves patient survival rates. The combination leverages the localized, tumor-targeting ability of radiation therapy and the systemic, immune-boosting effects of immunotherapy, potentially overcoming the limitations inherent in each treatment modality when used separately. This integrative approach is especially promising in addressing the complex tumor microenvironment of HCC. However, the treatment landscape is nuanced, with challenges such as patient-specific response variability and potential resistance to therapies. Future research directions should focus on refining these combination strategies, tailoring them to individual patient profiles, and understanding the underlying mechanisms that govern the interaction between immunotherapy and radiation therapy. Such advancements could significantly improve HCC management, setting new standards for patient care and treatment efficacy.

## 1. Introduction

Globally, liver cancer ranks as the seventh most frequently diagnosed cancer and stands as the third leading cause of cancer-related mortality [1]. Hepatocellular carcinoma (HCC) represents the predominant form of primary liver cancer, accounting for up to 85% of all cases of liver cancer [1]. The multifactorial etiology of HCC, encompassing viral hepatitis, alcoholic liver disease, and non-alcoholic fatty liver disease, contributes to its complexity and the challenges encountered in its management [2].

Traditional therapeutic avenues such as surgical interventions, transplantation, and locoregional therapies offer substantial benefits in the early stages of HCC. However, their applicability diminishes in advanced, metastatic, or recurrent diseases, necessitating the exploration of novel and effective treatment strategies [3]. Immunotherapy has surfaced as a beacon of hope, unveiling new horizons in the therapeutic landscape of HCC. By harnessing the body’s immune system, immunotherapy, encompassing immune checkpoint inhibitors (ICIs) and adoptive cell therapies, aims to bolster the immune response against the tumor, marking a significant advancement in the battle against HCC [4]. Radiation therapy continues to hold its ground as a pivotal element in the oncological arsenal. It is utilized for its capacity to exert cytotoxic effects on tumor cells, facilitating local control and palliation in HCC [5]. The integration of immunotherapy and radiation therapy presents a novel paradigm, suggesting a symbiotic relationship that could potentiate therapeutic outcomes. This amalgamation aims to capitalize on the strengths of each modality, fostering a conducive environment for enhanced tumor control and improving survival outcomes [5].

This review aspires to navigate through the intricate pathways of interaction between immunotherapy and radiation therapy in the context of HCC. It seeks to provide a comprehensive review of the preclinical and clinical data related to the combination of ICI and radiation therapy, addressing the expected challenges and future direction of this combination approach.

## 2. Overview of Current Management of HCC: Focus on ICI and Radiation Therapy

The treatment of HCC is guided by the Barcelona Clinic Liver Cancer (BCLC) staging system, which incorporates tumor burden, liver function, and patient performance status [6]. Most patients present with BCLC stage B or later, where curative treatments like resection, ablation, or transplantation are not feasible. Trans-arterial chemoembolization (TACE) only applies to a limited number of BCLC B-stage patients with specific criteria and is often used as a bridge to transplantation. However, many patients may not qualify for transplantation due to disease progression or liver function limitations [7,8,9]. The 2022 BCLC guidelines introduced updates for systemic therapies in advanced-stage HCC (BCLC C) [10].

### 2.1. Immunotherapy in HCC

Immunotherapy has marked a groundbreaking phase in treating liver cancer, introducing innovative strategies that enhance the body's own immune system to target and destroy cancer cells effectively [11]. At the heart of this advancement are treatments known as immune checkpoint inhibitors, including PD-1 and CTLA-4 blockers. These therapies work by boosting the immune system's ability to detect and fight cancer cells, significantly improving the chances of eliminating the disease [12,13].

The advent of ICI has significantly enhanced the management of HCC, particularly in the context of BCLC stages B-C and unresectable/metastatic disease. Initially, monotherapies targeting the programmed cell death protein 1 (PD-1), such as pembrolizumab and nivolumab, demonstrated potential but exhibited relatively limited clinical benefit when employed as later-line therapies following progression or intolerance to sorafenib [14,15,16]. Notably, two recent pivotal studies, IMBRAVE150 (NCT03434379) and HIMALAYA (NCT03298451), have demonstrated promising clinical benefits associated with the combination approach involving ICIs [12,13,17]. A phase 3 randomized clinical trial (RCT), IMBRAVE150, assessed the ICI and anti-angiogenesis combination approach, employing atezolizumab (anti-PD-L1) and bevacizumab (anti-VEGF) in treatment-naive advanced/metastatic HCC. Similarly, phase 3 RCT HIMALAYA investigated the dual checkpoint inhibitor approach with durvalumab (anti-PD-L1) and tremelimumab (anti-CTLA4) as the frontline treatment for advanced/metastatic HCC. Both trials have demonstrated promising efficacy with each combination approach, showcasing superior survival benefits compared to the historical first-line therapy, sorafenib. Currently, these two ICI based regimens are considered the standard front-line therapy for advanced/metastatic BCLC stage B-C HCC [10].

#### Artificial Intelligence-Based Pathology as a Biomarker for Immunotherapy in HCC

Innovative strides in HCC treatment have been made through the application of artificial intelligence (AI) in pathology, presenting a novel approach to predicting treatment sensitivity. A groundbreaking multicenter retrospective study introduces an AI model, the atezolizumab–bevacizumab response signature (ABRS-P), designed to predict sensitivity to atezolizumab–bevacizumab treatment in patients with hepatocellular carcinoma HCC. This AI model, trained on data from The Cancer Genome Atlas and validated through independent patient series, marks a significant advance in personalized cancer therapy. The study’s key outcomes demonstrate the ABRS-P model’s effectiveness in identifying patients likely to benefit from atezolizumab–bevacizumab based on the correlation between model predictions and actual progression-free survival rates. Patients with ABRS-P-high tumors exhibited notably longer median progression-free survival, highlighting the model’s potential as a reliable biomarker for treatment sensitivity. Moreover, the integration of spatial transcriptomics offered insights into the molecular characteristics associated with treatment response, illustrating the model’s ability to uncover biological mechanisms underlying HCC [18].

### 2.2. Radiation Therapy in HCC

The landscape of HCC management is multifaceted and marked by a diverse array of locoregional therapy (LRT) options, each catering to different stages of tumor progression. Radiation therapy has emerged as a crucial modality in HCC’s comprehensive management strategy, leveraging high-energy rays to precisely target and eliminate cancer cells, contributing significantly to local control and palliative care objectives. Traditionally, the application of external beam radiotherapy (EBRT) in managing HCC has been constrained due to technological hindrances and apprehensions regarding potential liver toxicity [19]. However, recent technological progress has facilitated the accurate administration of high-intensity radiation doses specifically to the liver in HCC cases, concurrently minimizing harm to the surrounding healthy tissues [20,21].

Rapid advancements in radiation therapy techniques, such as Stereotactic Body Radiation Therapy (SBRT), Selective Internal Radiation Therapy (SIRT), and External Beam Radiation Therapy (EBRT), represent a significant evolution in oncology that directly impacts HCC treatment. SBRT, capable of delivering high doses of potent radiation over a handful of treatment sessions without harming surrounding tissue, has shown exceptional efficacy for small, well-defined liver tumors. Similarly, SIRT, or radioembolization, which combines the embolic effect with targeted radiation delivery by directly injecting radioactive microspheres into the blood supply of the tumor, offers a therapeutic option for HCC patients with few alternatives. These innovations underscore a critical shift toward a highly patient-centric approach in the treatment of cancer, including HCC, where minimizing damage to healthy liver tissue is paramount [22,23].

For initial-stage HCC, the emphasis has predominantly been on ablation therapies. However, in the intermediate stages and selected cases of advanced-stage HCC, a spectrum of strategies comes into play, such as TACE, trans-arterial radioembolization (TARE), radiofrequency ablation (RFA), microwave ablation (MWA), and SBRT [24,25,26]. The advent of SBRT and proton therapy, in particular, has enhanced the precision and effectiveness of radiation delivery, minimizing unintentional harm to surrounding healthy tissues and offering a testament to the ongoing innovation within EBRT modalities such as Intensity-Modulated Radiation Therapy (IMRT) and Image-Guided Radiation Therapy (IGRT) [27,28].

The clinical panorama of radiation therapy in treating HCC is in a state of constant flux, enriched by recent research revelations elucidating its effectiveness and prospective applications. Contemporary studies have underscored the auspicious results linked with radiation therapy, reflecting enhancements in overall survival and a decline in recurrence rates. Such favorable outcomes permeate various therapeutic scenarios, where radiation therapy is either utilized as an isolated modality or is integrated synergistically with alternative interventions, like surgery or systemic therapies, to bolster treatment efficacy [29].

## 3. Current Limitations of ICIs and Radiation Therapy in HCC

As previously indicated, ICI has brought successful improvement in the management of HCC, and currently, ICI-based regimens are considered the standard front-line therapy for unresectable/metastatic HCC. However, only 15–30% of HCC patients have a durable response to these ICI regimes, and 20–40% of patients fail to respond [12,13,17]. A recent post hoc biomarker analysis from GO30140 or IMbrave150 studies pool by Zhu and colleagues suggested several potential predictive biomarkers [30], but these need external validation ideally by biomarker-driven prospect study to establish their roles. Currently, there is no reliable predictive biomarker for ICI in HCC to drive clinical decisions. There are vigorous ongoing efforts in CAR-T cell therapy and targeting other checkpoint molecules such as TIM-3, LAG-3, and TIGIT, which might broaden the range of ICI-based treatments for HCC. Further research might also pave the way for small-molecule inhibitors targeting the PD-1/PD-L1 pathway, offering an alternative with greater oral bioavailability, enhanced anti-tumor efficacy, and reduced toxicity [31].

Radioresistance in HCC involves multiple factors, including genetic mutations in DNA repair genes and altered signaling pathways like PI3K/AKT/mTOR and RAS/RAF/MEK/ERK. Hypoxia within tumors upregulates hypoxia-inducible factors (HIFs), leading to enhanced DNA repair and reduced apoptosis. The tumor microenvironment also contributes to resistance by secreting protective cytokines and growth factors. Strategies to overcome this resistance include targeted therapies, hypoxia-modifying agents, and epigenetic drugs, which show promise in enhancing the efficacy of radiotherapy in HCC [32,33]. The forefront of innovation is the combination of radiation therapy with other therapeutic methods. A notable area of exploration is its integration with immunotherapeutic agents, exemplified by studies investigating the synergy between radiation therapy, regorafenib, and PD-1 inhibitors, unveiling promising strides in therapeutic efficacy and manageable toxicity profiles in advanced HCC stages [34].

## 4. Combination of Radiotherapy and Immunotherapy

The dynamic interplay between immunotherapy and radiation therapy is carving a novel trajectory in the therapeutic management of cancer. The radioimmunotherapy combination has been meticulously explored in the field of oncology, including HCC, based on the multiple synergistic/complementary mechanistic rationales of these two anti-cancer modalities, namely radiation and immunotherapy.

### 4.1. Rationale of Radioimmunotherapy

Building on our previous discussion, radiation therapy significantly influences both the tumor and the host’s immunological microenvironments [35]. The response to radiation therapy notably includes the increased expression of MHC class I molecules on tumor cells, enhancing their visibility and recognition by cytotoxic CD8 T cells, and promoting intra-tumoral infiltration of cytotoxic T cells [36,37]. Additionally, dendritic cells, central to antigen presentation to T cells, are activated by radiation therapy. This activation involves the release of pro-inflammatory cytokines and the upregulation of co-stimulatory molecules, leading to cancer cell death and promoting the unveiling of neoantigens [38,39]. In addition, recent research has revealed that DNA damage caused by radiation therapy can induce a high mutational and neoantigen burden, which, in turn, promotes tumor death via cytotoxic T cell activity [40].

**Figure 1:**
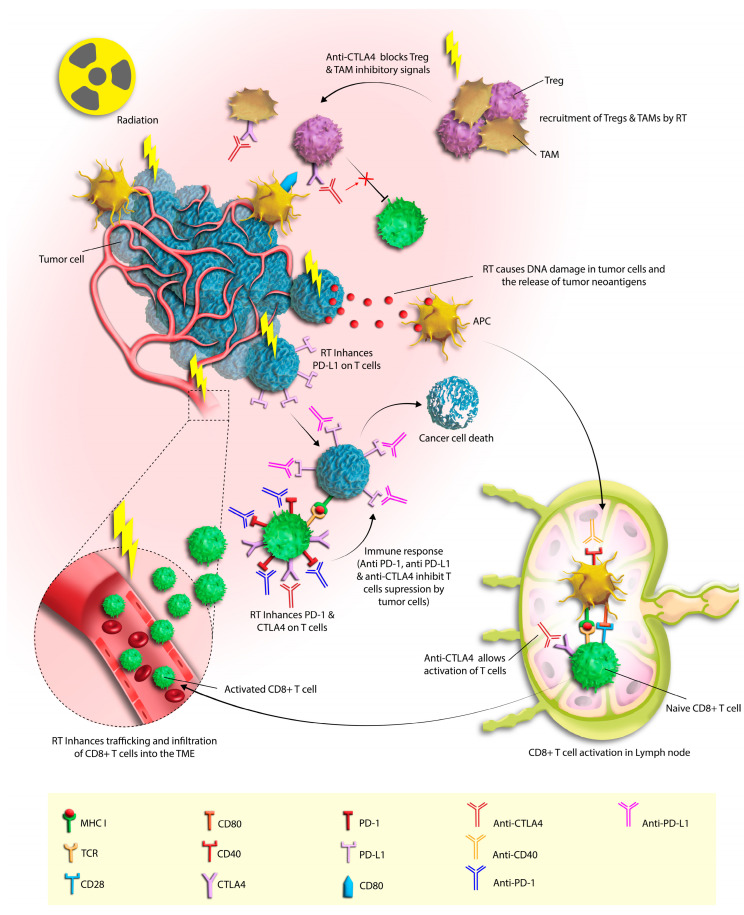
The synergistic effects of radiotherapy (RT) and immune checkpoint inhibition in hepatocellular carcinoma (HCC). RT initiates a potent anti-tumor immune response by enhancing antigen presentation and T cell activation, facilitating the recognition and destruction of tumor cells. Concurrently, RT may induce an immunosuppressive environment characterized by the recruitment of regulatory T cells (Tregs) and tumor-associated macrophages (TAMs), as well as upregulation of inhibitory checkpoint molecules PD1, PD-L1, and CTLA4 on immune and tumor cells. The administration of checkpoint inhibitors—anti-PD-1, anti-PD-L1, and anti-CTLA-4—acts to mitigate these immunosuppressive signals, thereby potentiating the cytotoxic activity of CD8+ T cells within the tumor microenvironment. This combination therapy paradigm aims to maximize tumor eradication while limiting tumor escape mechanisms.

Transitioning to the role of ICIs, these treatments disrupt specific cellular interactions, notably between PD-1 and PD-L1 or CTLA-4 and B7-1 (CD80)/B7-2 (CD86) [41]. This disruption facilitates T-cell activation with anti-tumor properties. Notably, when ICIs are paired with radiation therapy, a synergistic effect occurs, amplifying T cell-mediated cytotoxicity through mechanisms like improved antigen presentation and recognition, the release of pro-inflammatory cytokines, and the generation of tumor-specific antigens or neoantigens [42]. Concurrently, studies reveal that radiation enhances PD-L1 expression in tumor cells via the IFN-γ/STAT3 pathway [43]. Another study delves into the realm of this interaction, exploring the modulation of the immune response following radiation therapy. Their research elucidates that radiation therapy can induce the upregulation of PD-L1 on tumor cells, which could potentially influence the effectiveness of PD-1-based immunotherapies. In their innovative approach, they utilize attenuated Salmonella carrying siRNA-PD-L1, demonstrating that this combination could enhance the anti-tumor effects of radiation therapy in HCC. This synergy is manifested through inhibited tumor cell proliferation, enhanced apoptosis, and stimulated immune cell infiltration and activation within tumor tissues [44]. These processes induced by radiation therapy not only augment the priming and activation of immune cells, but also enhance the overall immune response against the tumor [45] (Figure 1).

These multiple mechanistic rationales well support a novel combination of radiation and immunotherapy, effectively merging the direct cytotoxic effects on the local tumor by radiation with the complementary effects of ICIs and TME modification by radiation therapy [43].

### 4.2. Optimal Timing of ICI during Radioimmunotherapy

The timing and sequencing of immunotherapy and radiation therapy are crucial for maximizing therapeutic synergy. For example, one study emphasized the significance of timing in administering ICIs in relation to whole-brain radiation therapy, suggesting that the sequence could influence clinical outcomes [46]. However, the ideal timing for administering ICIs in relation to radiation therapy—whether concurrently, prior to, or following radiation—remains unclear, as evidenced by the range of approaches observed in various animal studies [47,48].

Research indicates that combining CTLA-4 blockade with radiation therapy may bolster T-cell activity in tumors and extend survival in mouse models, as evidenced by two studies with differing administration sequences: one applied anti-CTLA-4 before radiation, while the other did the opposite. Additionally, a study by Young and colleagues found that administering anti-CTLA-4 before radiation was more effective, possibly due to reduced regulatory T cells. In a different approach, administering anti-OX40 one day post-radiation proved most effective in enhancing antigen presentation. However, the ideal timing for the PD-L1 blockade remains contested. While one study advocates for radiation before immunotherapy, another suggests that simultaneous treatment, rather than sequential, yields better survival outcomes [47,49].

Administering immunotherapy before radiation therapy aims to prime the immune system, enhancing the recognition of tumor antigens released during subsequent radiation therapy. Post-radiation administration of immunotherapy is also a focal point of research. Radiation therapy can modulate the tumor microenvironment, enhancing the expression of molecules such as PD-L1, thus providing a rationale for the subsequent introduction of immunotherapeutic agents like PD-1/PD-L1 inhibitors. A recent study demonstrated that combining siRNA-PD-L1 post-radiation could effectively enhance the anti-tumor effect, inhibiting tumor cell proliferation and stimulating immune cell infiltration [44].

In addition, the relative immunogenicity of different RT dosages, including hypofractionation versus conventional fractionation, is under investigation [50,51]. The determination of optimal dosage, fractionation, and sequencing of RT and immunotherapy is an area of ongoing research in HCC, influenced by factors like tumor size, location, and liver function.

### 4.3. Pathological Aspects of Hepatocellular Tumors Expressing PD-L1

PD-L1 significantly influences HCC progression by enabling immune evasion, which adversely affects patient outcomes. It mediates immunosuppression primarily through the inhibition of T-cell activity, promoting tumor growth. The regulation of PD-L1 expression is complex, involving pathways like IL-6/JAK/STAT3, which are pivotal for maintaining an immunosuppressive tumor microenvironment. Additionally, the tumor microenvironment itself, through interactions with tumor-associated macrophages and the PKM2-STAT1 pathway, can further stimulate PD-L1 expression. These insights suggest that immunotherapeutic strategies targeting not only PD-L1 but also these regulatory pathways could be crucial for reversing immune resistance and enhancing therapy effectiveness [52,53].

### 4.4. Strategies for Minimizing Hepatotoxicity in Combined Immunotherapy and Radiotherapy

The treatment of HCC through the synergistic use of immunotherapy and radiotherapy underscores the essential requirement to reduce hepatotoxicity, given the liver’s particular sensitivity to radiation. Recent innovations highlight a variety of tactics designed to lower this hazard, including the adoption of precision radiotherapy methods such as IMRT and SBRT. These techniques focus on accurately targeting the tumor masses while preserving the integrity of the surrounding healthy liver tissue. Enhancing the effectiveness of immunotherapy by selecting compounds that exert minimal hepatic side effects and scheduling their application in synchronization with radiotherapy serves as a further measure to curb hepatotoxicity. A critical component of this strategy is the ongoing assessment of liver function, enabling adjustments in dosage or temporary halts in treatment in response to the specific risk profile of each patient, thereby averting significant hepatic injury. The preventive employment of drugs aimed at safeguarding liver health adds another defensive layer against hepatotoxicity. Prioritizing patient evaluation and risk categorization before initiating therapy is crucial for tailoring treatment protocols that minimize negative outcomes. Beyond merely aiming to reduce liver toxicity, these approaches seek to amplify the combined effect of radiotherapy and immunotherapy. By refining radiation exposure, utilizing radioprotective agents, and exploiting precise targeting practices, this holistic treatment methodology aspires to bolster the reciprocal action between radiotherapy and immunotherapy. It achieves this by altering the TME to enhance the potency of ICIs, demanding a thorough grasp of the TME’s dynamic interrelations. The primary aim is to advance patient results by balancing the maximization of therapeutic efficiency against the minimization of adverse effects, requiring a collaborative approach to adeptly manage the complex dynamics within the TME [36,54].

### 4.5. Abscopal Effect and Its Implications for HCC

The abscopal effect involves systemic radiation-induced anti-tumor effects and is translated locally, representing a very promising therapeutic avenue for HCC patients which potentially exploits the immune system to recognize and destroy cancerous cells that are remote from the site of initial intervention, offering a complement to conventional therapies and a truly novel approach to management of this daunting neoplasm. Key mechanisms proposed are the release of tumor antigens following radiation-induced cell death, in turn leading to activation of T cells systemically when presented by antigen-presenting cells. This implies the ability to target not only the irradiated tumor but also distant metastases, indicating this to be a hopeful strategy for HCC, which so far has poor prognosis and limited therapy [55,56]. This phenomenon underscores the synergistic potential of combining radiotherapy with immunotherapy, suggesting that radiotherapy may target the local tumor and induce systemic immune responses beneficial for overall treatment outcomes. In the context of HCC, liver-directed combined radiotherapy as a downstaging strategy for liver transplantation has demonstrated favorable oncologic outcomes [57].

Clinical application of the abscopal effect in HCC still lies in its infancy, with a long way to go, although the continuous investigations are aimed at the optimization of radiation protocols and combinations with immunotherapies, which would reliably induce the phenomenon in practice. Further study on the molecular and immunological profile of HCC is critical in tailoring treatment strategies, based on exploiting the abscopal effect with hope for overall improvements in the management of patients [58,59].

### 4.6. Clinical Trials Investigating Radioimmunotherapy in Hepatocellular Carcinoma

Building upon the aforementioned rationale for radioimmunotherapy and the well-established roles of radiotherapy and ICI in HCC, the combination approach of radiation therapy and ICI has been under investigation in multiple clinical trials.

Incorporating specific genetic markers and biomarkers into the selection criteria for clinical trials investigating radioimmunotherapy in HCC can significantly refine patient selection and treatment outcomes. Recent studies highlight the potential of various genetic alterations and biomarkers in predicting responses to treatment. For example, alterations in the TERT promoter and mutations in genes such as TP53, CTNNB1, and the presence of specific non-coding RNAs have been associated with HCC progression and response to therapies. Additionally, the expression of PD-L1, a key marker in immunotherapy response, and serum proteins such as AFP (alpha-fetoprotein), have been explored for their predictive value in treatment efficacy. The integration of these genetic markers and biomarkers in trial design could enable more personalized and effective radioimmunotherapy strategies for HCC patients [60,61,62].

A phase 1 trial investigated the effectiveness and safety of combining SBRT with immunotherapy for treating advanced or inoperable HCC. Participants underwent SBRT and were then treated with either nivolumab alone or in combination with ipilimumab. The study’s primary concern was identifying dose-limiting toxicities within a 6-month period following SBRT. The findings revealed a more favorable outcome in patients treated with nivolumab and ipilimumab, showing higher response rates, extended progression-free, and overall survival [63]. A phase 2 single-arm study (START-FIT, NCT03817736) evaluated sequential TACE and SBRT followed by avelumab (anti-PDL1) in locally advanced HCC, with the rate of amenable to curative treatment as the primary endpoint. Thirty-three patients were included, with 36% in BCLC stages A-B and 64% in stage C without extrahepatic disease. The radioimmunotherapy strategy yielded promising conversion rates (55%) and radiographic complete response (42%) without demonstrating any new safety concerns [64]. Another phase 2 trial, CA 209-678 (NCT03033446), assessed Y90-radioembolization followed by nivolumab in 36 patients with advanced HCC. Among these patients, 36% had extrahepatic spread, and 44% had disease outside Y90-radioembolization fields. The primary endpoint was evaluated using the Simon two-stage design, targeting an ORR of 41%. The ORR was 30.6%, failing to meet the pre-specified target ORR. However, the ORR in patients without extrahepatic disease was at 43.5%. Conversely, patients with extrahepatic disease showed a limited ORR of 7.7%. Treatment-related grade 3–4 adverse events or serious adverse events occurred in 14% of patients, but the overall combination was well-tolerated without any treatment-related deaths [65]. These findings suggest that further investigation of this radioimmunotherapy strategy is warranted, especially in patients with refractory or ineligible BCLC B disease for TACE and those with BCLC C disease without extrahepatic spread.

Table 1 below lists the ongoing trials incorporating RT and ICIs in HCC. This table provides a snapshot of current research endeavors, showcasing the diverse approaches adopted to enhance HCC treatment.

The trials listed in Table 1 represent a significant push toward innovation in HCC treatment. The results of this series of ongoing clinical trials elucidate multiple questions, including (1) the overall role of radiation therapy and ICI combination in HCC, (2) the optimal timing of ICI administration within the peri-radiation context, (3) the proper types and doses of radiation therapy, and (4) the ideal clinical setting in the context of HCC for this combination approach. The success of these trials could lead to changes in clinical practice, offering more effective and personalized treatment options for HCC patients.

## 5. Challenges and Future Directions

The journey towards integrating immunotherapy and radiation therapy in treating HCC is laden with challenges. As we navigate this complex terrain, understanding these challenges and envisioning future directions becomes paramount.

### 5.1. Balancing Efficacy and Safety: A Primary Challenge

While promising, the confluence of immunotherapy and radiation therapy brings forth unique challenges. Balancing the efficacy of these potent treatments with the safety of patients is a delicate act, especially considering the potential for severe adverse events. The heterogeneous nature of HCC further complicates patient selection, as responses to combined therapy can vary significantly. Additionally, the high cost and limited accessibility of advanced immunotherapies and radiation techniques pose significant barriers, especially in resource-limited settings.

### 5.2. Challenges in Current Therapeutic Approaches

Although preclinical studies have shown promising results [66], to our knowledge, there are not enough published prospective clinical trials on the combined use of RT and HCC, except for a few limited series demonstrating encouraging clinical effectiveness [63,67].

Given that this area of research is still in its early stages, most studies have indicated promising effectiveness and an acceptable toxicity profile for patients treated with a combination of RT and ICI; they have yet to determine the optimal RT dose, fractionation schedule, and sequencing with ICI. Factors such as the type of cancer, the specific ICI used, and the tumor’s histological and mutational characteristics greatly influence these variables [68]. Moreover, a dose range of 8–10 Gy RT administered in one to three fractions is proposed to induce an abscopal effect [68,69], and the radio-sensitivity of nearby vasculature, the toxicity profile, and the identification of pro-immunogenic signatures following RT are critical considerations for optimizing protocols [70].

Another formidable hurdle is the resistance to therapy, either inherent or acquired, which can impede the success of these treatments. Numerous unresolved questions remain in the field, yet the combination of radiation therapy and immunotherapy holds substantial promise due to their synergistic effects. Thus, further research to address these issues and advance the development of radiation immunotherapy is of paramount importance.

### 5.3. Envisioning Future Directions

The future of HCC treatment lies in the realm of personalized medicine. Genomics and biomarker research advancements hold the key to tailoring therapy based on individual patient profiles, aiming to maximize efficacy while minimizing toxicity. Exploring various combination and sequencing strategies through ongoing and future trials is critical, including the convergence of radiation therapy with immunotherapy, which could enhance therapeutic outcomes in cancer treatment, including HCC [71].The complexity of combining immunotherapy and radiation therapy necessitates an interdisciplinary approach, fostering collaboration across various fields such as oncology, radiology, immunology, and pharmacology. As we stand at the crossroads of a potential paradigm shift in HCC treatment, embracing these challenges and future directions is pivotal. Through this concerted effort, we can harness the full potential of combining immunotherapy and radiation therapy, ultimately leading to a new standard of care that offers improved outcomes for patients battling this formidable disease.

## 6. Conclusions

The exploration of combining immunotherapy and radiation therapy in HCC treatment is a rapidly evolving and promising area of oncology. This approach marks a significant shift in the therapeutic landscape, offering new hope and possibilities for effective HCC management.

Clinical trials, such as those investigating the synergistic effects of durvalumab with SBRT, nivolumab with radiotherapy, and the innovative combination of atezolizumab, bevacizumab, and SBRT, are at the forefront of this evolution. Each study contributes critical insights into optimizing treatment strategies, aiming to enhance the efficacy and precision of HCC therapies.

Despite the potential of these combined modalities, the journey is fraught with challenges. Managing the unique adverse events, addressing the variability in patient responses, overcoming resistance to therapies, and ensuring the accessibility and affordability of treatments are key hurdles that need to be addressed. The future direction of HCC treatment is steering towards personalized medicine, where therapies are tailored to individual patient profiles, and novel combination and sequencing strategies are continuously explored.

The ongoing research and clinical trials are vital in overcoming these challenges, setting the stage for more effective, personalized, and accessible treatment options for HCC. As the medical community eagerly anticipates the results of these studies, there is growing optimism about the potential to improve patient outcomes and quality of life significantly.

In summary, the integration of immunotherapy and radiation therapy in HCC signifies a dynamic and transformative field filled with both opportunities and challenges. The continued advancements in this area hold the promise of establishing new standards of care, ultimately leading to enhanced treatment outcomes for patients facing this challenging disease.

## Figures and Tables

**Table 1:** Ongoing trials of RT + ICIs in HCC *.

NCI ID (Trial)	Phase	Eligibility	Type of RT	Type of ICI	Design	Target Enrollment	Primay Endpoint	Status	Estimated Completion
NCT04857684	1	Resectable Child–Pugh: A	SBRT	Atezo–Bev (anti-PDL1/anti-VEGF)	SBRT -> 2 cycles of Atezo–Bev -> Surgery	20	G3-4 TRAE rate	Recruiting	31 December 2024
NCT05286320	1/2	Disease with PVI Child–Pugh: A	SBRT	Pembrolizumab + Lenvatinib (antiPD1/TKI)	Pembrolizumab + Lenvatinib SBRT during C2 of pembrolizumab	27	Phase1: DLT Phase2: ORR	Not Yet Recruiting	30 September 2026
NCT05625893 (PORTAL)	2	Disease with PVI Child–Pugh: A	Proton radiotherapy	Atezo–Bev (anti-PDL1/anti-VEGF)	Atezo–Bev PBT 1 wk after C2 Atezo–Bev	63	PFS	Recruiting	31 December 2025
NCT05339581 (iPLENTY-pvtt)	N/A	Disease with PVI Child–Pugh: 7 or less	IMRT	anti-PD1 + Lenvatinib (TKI)	Anti-PD1 + Lenvatinib + IMRT (C3 of anti-PD1) or Anti-PD1 + Lenvatinib	78	ORR	Not Yet Recruiting	31 May 2024
NCT06040177	2	Unresectable with PVI BCLC: Stage C Child–Pugh: 7 or less	SBRT	Cadonilimab (anti-PD1/CTLA4)	Renvatinib SBRT -> Cadonilimab	30	ORR	Recruiting	1 February 2025
NCT04913480	2	Unresectable, non-metastatic BCLC: Stage C or earlier Child–Pugh: 7 or less	SBRT	Durvalumab (anti-PDL1)	Durvalumab SBRT 1 wk after 1st Durvalumab	37	PFS at 1 year	Recruiting	31 December 2024
NCT03942328	1/2	Unresectable, non-metastatic BCLC: Stage C or lower Child–Pugh: A	EBRT	Autologous Dendritic Cells + Atezo–Bev (anti-PDL1/anti-VEGF)	EBRT (1–3 wks) -> Autologous Dendritic Cells + Atezo–Bev	54	DLT PFS at 2 years	Recruiting	31 August 2027
NCT04988945	2	Non-metastatic Child–Pugh: 7 or less	SBRT	Durva–Treme (anti-PDL1/CTLA4)	TACE & SBRT -> Durva–Treme	33	Downstaging for resection rate	Recruiting	1 December 2024
NCT05488522	1	Non-metastatic Child–Pugh: 7 or less	SBRT	Atezo–Bev (anti-PDL1/anti-VEGF)	Atezo–Bev SBRT on wk2	18	DLT	Recruiting	31 December 2024
NCT06133062 (ProtonAB)	2	Non-metastatic BCLC: Stage B-C Child–Pugh: A	Proton radiotherapy	Atezo–Bev (anti-PDL1/anti-VEGF)	Proton radiotherapy with Atezo–Bev	45	PFS	Recruiting	30 September 2028
NCT05992220 (ALERT-HCC)	2,RCT	Non-metastatic with vascular invasion Child–Pugh: A	EBRT	Atezo–Bev (anti-PDL1/anti-VEGF)	Atezo–Bev + EBRT after C1D2 of Atezo–Bev vs. Atezo–Bev w/o EBRT	138	PFS	Recruiting	31 March 2026
NCT05096715	1	Non-metastatic BCLC: Stage B-C Child–Pugh: A	SBRT	Atezo–Bev (anti-PDL1/anti-VEGF)	SBRT + Atezo–Bev -> Atezo–Bev	20	DLT	Not Yet Recruiting	1 January 2026
NCT05377034 (STRATUM)	2, RCT	Non-metastatic Child–Pugh: A	Radioembolization (yttrium-90)	Atezo–Bev (anti-PDL1/anti-VEGF)	Radioembolization -> Atezo–Bev vs. Atezo–Bev	176	ORR at 1 year	Recruiting	1 November 2025
NCT03316872	2	Advanced/Metastatic Child–Pugh: A	SBRT	Pembrolizumab (anti-PD1)	Pembrolizumab SBRT on C1D2 of Pembrolizumab	30	ORR	Recruiting	N/A
NCT04430452	2	Advanced/Metastatic Child–Pugh: 8 or less Progression on anti-PD1/PDL1	Hypofractionated radiotherapy	Durva–Treme (anti-PDL1/CTLA4)	Hypofractionated RT -> Durvalumab or Durva–Treme	21	ORR	Recruiting	28 February 2027
NCT05396937	2	Metastatic Child–Pugh: 7 or less	SBRT	Atezo–Bev (anti-PDL1/anti-VEGF)	Atezo–Bev SBRT 1–2 wks after C1 Atezo–Bev	42	ORR	Recruiting	N/A
NCT05809869	2	Metastatic	Radioembolization (yttrium-90)	Durva–Treme (anti-PDL1/CTLA4)	Durva–Treme Radioembolisation on wk 2	25	ORR	Recruiting	31 December 2025

* This information is available on https://clinicaltrials.gov/ (accessed on 14 January 2024). Abbreviations: Atezo–Bev, Atezolizumab plus bevacizumab; C, Cycle; DLT, Dose-limiting toxicity; Durva–Treme, Durvalumab plus tremelimumab; EBRT, External beam radiation therapy; G, Grade; ICI, Immune checkpoint inhibitor; IMRT, Intensity-modulated radiotherapy; N/A, Not available; ORR, Objective response rate; PBT, Proton beam radiotherapy; PFS, Progression-free survival; PVI, Portal vein invasion; RCT, Randomized clinical trial; RT, Radiation Therapy; SBRT, Stereotactic body radiotherapy; TACE, Transarterial chemoembolization; TKI, Tyrosine kinase inhibitor; TRAE, Treatment-related adverse effect; Wk, Week.
